# Assessment of Mental Health of High School Students During Social Distancing and Remote Schooling During the COVID-19 Pandemic in Austria

**DOI:** 10.1001/jamanetworkopen.2021.14866

**Published:** 2021-06-28

**Authors:** Christoph Pieh, Paul L. Plener, Thomas Probst, Rachel Dale, Elke Humer

**Affiliations:** 1 Department for Psychotherapy and Biopsychosocial Health, Danube University, Krems, Austria; 2 Department of Child and Adolescent Psychiatry, Medical University of Vienna, Vienna, Austria; 3 Department of Child and Adolescent Psychiatry and Psychotherapy, University of Ulm, Ulm, Germany

## Abstract

This cross-sectional study assesses multiple aspects of mental health among high school students during the COVID-19 pandemic in Austria.

## Introduction

To get the COVID-19 pandemic under control, many countries have imposed lockdown measures or remote schooling. This study assessed mental health in high school students aged 14 to 20 years after 1 semester of attending school remotely and almost a year of social distancing in Austria.

## Methods

This cross-sectional study was approved by the local Ethics Committee and was conducted according to the guidelines of the Declaration of Helsinki.^1^ This study follows the American Association for Public Opinion Research (AAPOR) reporting guideline.

This cross-sectional study was supported by the Austrian Federal Ministry of Education, Science and Research, which informed and invited all schools to participate. Adolescents were recruited to be representative by region. Participants had to agree to the data protection declaration to start the survey, which served as electronic informed consent. Well-being (assessed with the World Health Organization–5 Well-being Index [WHO-5]^2^), depressive symptoms (assessed with the Patient Health Questionnaire-9 [PHQ-9]^3^), anxiety symptoms (assessed with the General Anxiety Disorder-7 [GAD-7]^4^), sleep quality (assessed with the Insomnia Severity Index [ISI]^5^), and disordered eating (assessed with the Eating Attitudes Test [EAT-8]^6^) were assessed via an online survey open from February 3 to February 28, 2021 (eMethods in the Supplement). Smartphone use and its association with mental health were analyzed using SPSS statistical software version 26 (IBM).

Descriptive statistics, *t* tests, χ^2^ tests, and univariate analysis of variance were computed. Effect sizes are shown as Hedge *g* or η^2^. *P* values were 2-tailed, and statistical significance was set at *P* = .05. Data were analyzed from March 1 to March 11, 2021.

## Results

A total of 3052 adolescents (mean [SD] 16.5 [1.4] years; 2139 [70.1%] girls; 508 students [16.6%] with migration background) participated in the study. A total of 1514 students (55.0%) exceeded the cutoff for clinically relevant depressive symptoms (ie, PHQ-9 score, ≥11), 1326 students (47.0%) had clinically relevant anxiety symptoms (ie, GAD-7 score, ≥11), 680 students (22.8%) had clinically relevant moderate insomnia (ie, ISI score, ≥15), and 1702 students (59.5%) had clinically relevant disordered eating behavior (ie, EAT-8 score, ≥2/3). Measures of psychological health by self-reported gender are summarized in Table 1. The prevalence of suicidal ideation (item 9 of the PHQ-9) within the last 2 weeks was 1016 students (36.9%), including 246 students (8.9%) with suicidal ideation nearly every day and 203 students (7.4%) with suicidal ideation more than half the days. Increased mobile phone use was associated with worse mental health (Table 2).

**Table 1.** Measures of Psychological Health by Gender

Measure	Total	Girls	Boys	Diverse^a^	Statistic	*P* value
WHO-5						
No.	3049	2137	856	56	F_2,3046_ = 64.80	
Score, mean (SD)	37.8 (20.9)	35.8 (19.7)	43.9 (22.4)	22.4 (18.2)	η^2^ = 0.041	<.001
PHQ-9						
No.	2752	1938	764	50	F_2,2749_ = 117.16	
Score						
Mean (SD)	11.9 (6.6)	12.8 (6.4)	9.2 (6.4)	18.2 (5.4)	η^2^ = 0.079	<.001
≥11, No. (%)	1514 (55.0)	1195 (61.7)	272 (35.6)	47 (94.0)	χ^2^_2,2752_ = 181.64	<.001
GAD-7						
No.	2821	1988	783	50	F_2,2818_ = 110.95	
Score						
Mean (SD)	10.3 (5.3)	11.1 (5.0)	8.1 (5.3)	14.2 (4.9)	η^2^ = 0.073	<.001
≥11, No. (%)	1326 (47.0)	1041 (52.4)	250 (31.9)	35 (70.0)	χ^2^_2,2821_ = 104.98	<.001
ISI						
No.	2988	2101	835	52	F_2,2985_ = 67.78	
Score						
Mean (SD)	10.3 (5.7)	11.0 (5.5)	8.4 (5.6)	12.2 (4.9)	η^2^ = 0.043	<.001
≥15, No. (%)	680 (22.8)	543 (25.8)	122 (14.6)	15 (28.8)	χ^2^_2,2988_ = 44.02	<.001
EAT-8						
No.	2862	2017	794	51	F_2,2859_ = 161.86	
Score						
Mean (SD)	3.37 (2.7)	3.89 (2.7)	2.0 (2.1)	4.0 (2.8)	η^2^ = 0.102	<.001
≥2, No. (%)	1702 (59.5)	1295 (64.2)	377 (47.5)	305 (58.8)	χ^2^_2,2862_ = 66.11	<.001

Abbreviations: EAT-8, Eating Attitudes Test; GAD-7, Generalized Anxiety Disorder-7; ISI, Insomnia Severity Index; PHQ-9, Patient Health Questionnaire-9; WHO-5, World Health Organization-5 Well-being Index.

^a^ Diverse indicates persons whose gender identity or gender expression does not conform to socially defined male or female gender norms.

**Table 2.** Measures of Psychological Health by Smartphone Use

Measure	Smartphone use, h/d						Statistic	*P* value
	<1	1-2	3-4	5-6	7-8	>8		
WHO-5								
No.	47	381	931	787	430	472	F_5,3042_ = 56.42	
Score, mean (SD)	48.5 (25.1)	45.3 (22.1)	42.8 (20.8)	37.1 (19.6)	31.2 (18.0)	28.2 (18.2)	η^2^ = 0.085	<.001
PHQ-9								
No.	45	343	843	717	383	421	F_5,2746_ = 67.01	
Score, mean (SD)	9.31 (7.4)	9.0 (6.1)	10.2 (6.2)	12.4 (6.2)	13.7 (6.1)	15.6 (6.6)	η^2^ = 0.109	<.001
GAD-7								
No.	45	349	863	745	389	430	F_5,2815_ = 37.65	
Score, mean (SD)	9.2 (6.8)	8.6 (5.2)	9.2 (5.0)	10.6 (5.1)	11.2 (5.0)	12.7 (5.2)	η^2^ = 0.063	<.001
ISI								
No.	47	374	914	778	417	458	F_5,2982_ = 40.64	
Score, mean (SD)	8.3 (7.0)	8.4 (5.7)	9.1 (5.4)	10.7 (5.4)	11.4 (5.4)	12.7 (5.6)	η^2^ = 0.064	<.001
EAT-8								
No.	45	356	877	753	396	435	F_5,2856_ = 12.88	
Score, mean (SD)	2.2 (2.3)	2.9 (2.6)	3.0 (2.6)	3.5 (2.7)	3.8 (2.7)	3.9 (2.8)	η^2^ = 0.022	<.001

Abbreviations: EAT-8, Eating Attitudes Test; GAD-7, Generalized Anxiety Disorder-7; ISI, Insomnia Severity Index; PHQ-9, Patient Health Questionnaire-9; WHO-5, World Health Organization-5 Well-being Index.

In Austria mental health is regularly assessed via the Health Behaviour in School-Aged Children study.^7^ Since the latest Health Behaviour in School-Aged Children study in 2018,^7^ mental well-being, as assessed using scores on the WHO-5, decreased from a mean (SD) score of 43.7 (19.8) to 35.8 (19.7) in girls (t_2136_ = −18.58; *P* < .001; *d* = −0.40) and from 53.1 (19.5) to 43.9 (22.4) in boys (*t*_855_ = −12.00; *P* < .001; *d* = −0.43). Life-satisfaction, measured with an 11-point Cantril ladder from 0, indicating the worst possible life, to 10, the best possible life, decreased from a mean (SD) of 7.1 (1.8) to 5.9 (2.0) in girls (*t*_2138_ = −27.91; *P* < .001; *g* = −0.62) and from 7.6 (1.6) to 6.3 (2.1) in boys (*t*_856_ = −17.83; *P* < .001; *d* = −0.66). Mobile phone use has increased 2-fold overall since 2018, with high users (ie, >8 h/d) increasing more than 4-fold (boys: χ^2^_5_ = 104.45; *P* < .001; girls: = χ^2^_5_ = 278.53; *P* < .001).

## Discussion

The findings of this cross-sectional study suggest that the COVID-19 pandemic was associated with impaired mental health. The mental well-being and life satisfaction in adolescents in Austria were significantly lower in 2021 compared with in 2018.^7^ Depressive symptoms, anxiety symptoms, insomnia, and disordered eating were significantly higher than prior to and in the beginning of the COVID-19 pandemic. Suicidal ideation among our study sample was significantly higher than in comparative studies, with approximately one-third of adolescents reporting suicidal thoughts. Smartphone use increased compared with 2018 rates and was significantly associated with mental health.

This study has some limitations. The cross-sectional design allows no causal conclusions. Additionally, owing to the online nature of the study, a self-selection bias toward higher participation of adolescents with a higher mental health burden is possible.

Our results suggest a high prevalence of mental disorders 1 year after the COVID-19 pandemic began in Austria. These findings highlight the need to implement health promotion and prevention strategies among adolescents.

## Notes

Supplement. eMethods.
