# Supplementary material for: Assessment of Mental Health of High School Students During Social Distancing and Remote Schooling During the COVID-19 Pandemic in Austria
Source: JAMA Netw Open. 2021 Jun 28;4(6):e2114866. doi: 10.1001/jamanetworkopen.2021.14866 (PMC8239947; doi:10.1001/jamanetworkopen.2021.14866)
Supplement: Supplement. — eMethods. [file jamanetwopen-e2114866-s001.pdf]

Supplemental Online Content

Pieh C, Plener PL, Probst T, Dale R, Humer E. Assessment of mental health of high school students during social distancing and remote schooling during the COVID-19 pandemic in Austria. *JAMA Netw Open*. 2021;4(6):e2114866. doi:10.1001/jamanetworkopen.2021.14866

eMethods.

This supplemental material has been provided by the authors to give readers additional information about their work.

© 2021 Pieh C et al. *JAMA Network Open.*

eMethods.

1. Restrictions and Remote Schooling in Austria

Since October 2020 high school took place mainly remotely. In this time, no team or indoor sport was allowed. In November 2020 and from December 2020 until January 2021 one was allowed to leave the house only in a few exceptions, such as meeting necessary basic needs of daily life, assistance for people in need or outdoor activities alone or with people from the same household. Starting on February 8, 2021 the strict lockdown was lifted, and high schools were allowed to reopen with classes in a shift system and extended protective measures such as weekly COVID-19 tests and wearing FFP2 face masks.

2. Measures

2.1. Well-being (WHO-5)

The WHO-5 questionnaire[1] measures well-being with five self-rating items rated on a six-point Likert scale. The score can range from 0 (absence of well-being) to 25 (maximal well-being). To translate into a percentage scale from 0 to 100 raw scores were multiplied by four. The German version of the WHO-5 has good psychometric properties in adolescents[2]. Cronbach's alpha was $\alpha = 0.85$ in the current sample.

2.2. Depressive symptoms (PHQ-9)

The depression module of the Patient Health Questionnaire (PHQ-9) was used to measure depressive symptoms[3]. The German version of the PHQ-9 is validated for adolescents[4]. The PHQ-9 contains nine self-rating items on a four-point scale ranging from 0 (“not at all”) to 3 (“nearly every day”) with a sum score from 0 to 27. The recommended cut-off score of greater-equal 11 points for detecting major depression in adolescents[5] was used to define clinically relevant depression. Cronbach's alpha was $\alpha = 0.88$ in the current sample. Item 9 of the PHQ-9 has been shown to be a robust and age-independent predictor of suicide attempts and deaths[6] and was therefore analyzed as a measure of suicidal ideation. This item asks “Over the last two weeks, how often have you been bothered by thoughts that you would be better off dead or of hurting yourself in some way?”. Response to this question can be coded in a binary way to identify the presence of any recent suicidal ideas within the last two weeks (presence of suicidal thoughts = item endorsed if response ranged from 1 to 3; no suicidal thoughts = item not endorsed if response was 0).

© 2021 Pieh C et al. *JAMA Network Open.*

2.3. Anxiety symptoms (GAD-7)

The German version of the Generalized Anxiety Disorder 7 scale (GAD-7) was used to measure anxiety symptoms[7]. It measures anxiety with 7 self-rating items on a four-point scale, from 0 to 3 (maximum score 21). Cut-off points ≥11 for moderate and ≥17 for severe anxiety symptom levels were suggested for adolescents[8]. The 11-point cut-off was used in the current study to define clinically relevant anxiety. Cronbach's alpha for anxiety was $\alpha = 0.88$ in the current sample.

2.4. Sleep quality (ISI)

The German version of the Insomnia Severity Index (ISI) is a validated screening instrument for insomnia in adolescents[9]. It measures sleep quality and insomnia on 7 self-reported items on a four-point scale ranging from 0 to 4. The sum scores range from 0 to 28 with a recommended cut-off score for clinical insomnia (moderate severity) greater-equal 15 points[10]. Cronbach's alpha was $\alpha = 0.80$ in the current sample.

2.5. Eating attitudes test (EAT-8)

Disordered eating was measured with a reliable, validated, 8 items self-rating instrument, EAT-8[11]. It offers a screening instrument to screen individuals at high risk of developing clinical eating disorders. The EAT-8 is a short version of the EAT-26[12] including 8 items in a dichotomized response format (1 = "I agree somewhat" and 0 = "I disagree somewhat"). Through the determination of a total score ranging from 0 to 8, the EAT-8 should classify into a low risk and a high-risk group. In this study, the more conservative cut-off points of 3 for female and 2 for male adolescents were used, as they were recommended for epidemiological purposes.[11] Cronbach's alpha was $\alpha = 0.84$.

2.6. Life satisfaction

Life satisfaction was measured with the Cantril ladder, an instrument that has been shown to be valid and reliable to assess overall mental well-being among adolescents[13]. Adolescents were asked to rate their life satisfaction on a 11-point scale ranging from (0) "the worst possible life" to (10) "the best possible life".

2.7. Migration status and smartphone usage

© 2021 Pieh C et al. *JAMA Network Open.*

Students were asked whether they or both parents were born abroad, to assess their migration status. Mobile phone use was queried identically as in the Health-Behaviour in School-aged Children-study (HBSC-study) conducted in Austria in 2018[14] in order to allow for comparison.

3. Completion rate

Following the American Association for Public Opinion Research (AAPOR) reporting guidelines for non-probability online samples,[15] we report that 81.1% of those who clicked on the survey link completed the entire survey, resulting in a 81.1% participation rate (ie, completion rate).[16]

References

1. Brähler E, Mühlan H, Albani C, Schmidt S. Teststatistische Prüfung und Normierung der Deutschen Versionen des EUROHIS-QOL Lebensqualität-index und des WHO-5 Wohlbefindens-index. *Diagnostica*. 2007;53(2):83-96. doi:10.1026/0012-1924.53.2.83

2. Allgaier AK, Pietsch K, Frühe B, Prast E, Sigl-Glöckner J, Schulte-Körne G. Depression in pediatric care: Is the WHO-Five Well-Being Index a valid screening instrument for children and adolescents? *Gen Hosp Psychiatry*. 2012;34(3):234-241. doi:10.1016/j.genhosppsych.2012.01.007

3. Spitzer RL, Kroenke K, Williams JBW. Validation and utility of a self-report version of PRIME-MD: The PHQ Primary Care Study. *JAMA*. 1999;282(18):1737-1744. doi:10.1001/jama.282.18.1737

4. Allgaier AK, Pietsch K, Frühe B, Sigl-Glöckner J, Schulte-Körne G. Screening for depression in adolescents: Validity of the patient health questionnaire in pediatric care. *Depress Anxiety*. 2012;29(10):906-913. doi:10.1002/da.21971

5. Richardson LP, McCauley E, Grossman DC, et al. Evaluation of the patient health questionnaire-9 item for detecting major depression among adolescents. *Pediatrics*. 2010;126(6):1117-1123. doi:10.1542/peds.2010-0852

6. Rossom RC, Coleman KJ, Ahmedani BK, et al. Suicidal Ideation Reported on the PHQ9 and Risk of Suicidal Behavior across Age Groups. *J Affect Disord*. 2017;215:77-84. doi:10.1016/j.jad.2017.03.037

7. Löwe B, Decker O, Müller S, et al. Validation and standardization of the generalized anxiety

© 2021 Pieh C et al. *JAMA Network Open.*

disorder screener (GAD-7) in the general population. *Med Care*. 2008;46(3):266-274. doi:10.1097/MLR.0b013e318160d093

8. Mossman SA, Luft MJ, Schroeder HK, et al. The Generalized Anxiety Disorder 7-item scale in adolescents with generalized anxiety disorder: Signal detection and validation. *Ann Clin Psychiatry*. 2017;29(4):227-234A.

9. Gerber M, Lang C, Lemola S, et al. Validation of the German version of the insomnia severity index in adolescents, young adults and adult workers: Results from three cross-sectional studies. *BMC Psychiatry*. 2016;16(1). doi:10.1186/s12888-016-0876-8

10. Morin CM, Belleville G, Bélanger L, Ivers H. The insomnia severity index: Psychometric indicators to detect insomnia cases and evaluate treatment response. *Sleep*. 2011;34(5):601-608. doi:10.1093/sleep/34.5.601

11. Richter F, Strauss B, Braehler E, Altmann U, Berger U. Psychometric properties of a short version of the Eating Attitudes Test (EAT-8) in a German representative sample. *Eat Behav*. 2016;21:198-204. doi:10.1016/j.eatbeh.2016.03.006

12. Garner DM, Bohr Y, Garfinkel PE. The Eating Attitudes Test: Psychometric Features and Clinical Correlates. *Psychol Med*. 1982;12:871-878. doi:10.1017/S0033291700049163

13. Jovanović V. The validity of the Satisfaction with Life Scale in adolescents and a comparison with single-item life satisfaction measures: a preliminary study. *Qual Life Res*. 2016;25(12):3173-3180. doi:10.1007/s11136-016-1331-5

14. Teutsch F, Ramelow D, Maier G, Felder-Puig R. Lineartabellen zu den Ergebnissen der HBSC-Studie 2018: Gesundheit und Gesundheitsverhalten von Schülern in Österreich. Bundesministerium; 2018.

15. American Association for Public Opinion Research. *Standard Definitions: Final Dispositions of Case Codes and Outcome Rates for Surveys*. 9th ed. American Association for Public Opinion Research; 2016.

16. Callegaro M, DiSogra, C. Computing response metrics for online panels. *Public Opinion Q.* 2008;72(5):1008-1032. doi:10.1093/poq/nfn065

© 2021 Pieh C et al. *JAMA Network Open.*
